# Corneal Densitometry After Small Incision Lenticule Extraction (SMILE) and Femtosecond Laser-Assisted LASIK (FS-LASIK): 5-Year Prospective Comparative Study

**DOI:** 10.3389/fmed.2020.521078

**Published:** 2020-11-06

**Authors:** Ruoyan Wei, Meiyan Li, Weiming Yang, Yang Shen, Yu Zhao, Dan Fu, Jianmin Shang, Jing Zhang, Joanne Choi, Xingtao Zhou

**Affiliations:** ^1^Department of Ophthalmology and Optometry, Eye and ENT Hospital, Fudan University, Shanghai, China; ^2^NHC Key Laboratory of Myopia, Fudan University, Shanghai, China; ^3^Shanghai Research Center of Ophthalmology and Optometry, Shanghai, China; ^4^Department of Ophthalmology, Children's Hospital of Fudan University, Shanghai, China; ^5^Department of Epidemiology and Biostatistics, School of Public Health, Fudan University, Shanghai, China; ^6^Department of Ophthalmology, Kresge Eye Institute, Wayne State University, Detroit, MI, United States

**Keywords:** corneal densitometry, small incision lenticule extraction (SMILE), femtosecond laser-assisted LASIK (FS-LASIK), myopia, pentacam scheimpflug imaging

## Abstract

**Purpose:** To investigate long-term changes in corneal densitometry (CD) following small incision lenticule extraction (SMILE) vs. femtosecond laser-assisted LASIK (FS-LASIK) in patients with myopia or myopic astigmatism.

**Methods:** Prospective analysis was performed in 66 eyes of 38 patients (13 males) who underwent SMILE and 54 eyes of 29 patients (5 males) who underwent FS-LASIK. In all patients, an ocular examination was performed preoperatively, and at 6–12 months and 5 years postoperatively. CD was obtained with the Pentacam Scheimpflug imaging system at the 0–2-mm, 2–6-mm, and 6–10-mm zones of the cornea at depth of anterior 120 μm, midcornea, and posterior 60 μm. Correlation analysis was performed between postoperative change in CD and other variables such as age, type of surgery, central corneal thickness, spherical equivalent, lenticule thickness/ablation depth, and changes in wavefront aberrations.

**Results:** At postoperative 6–12 months, a significant reduction at several corneal zones in the FS-LASIK cohort (*P* < 0.05) was observed. In the SMILE cohort, no significant change in CD relative to baseline was observed. However, at 5 years postoperatively, in both groups, a significant decrease in CD was observed in three zones of three layers (all *P* < 0.001). The change in CD was similar between groups at postoperative 6–12 months, but at 5 years the magnitude of change was significantly smaller in SMILE than FS-LASIK in the anterior and central layers (all *P* < 0.05).

**Conclusion:** CD with the Scheimpflug imaging system showed a significant decrease at 5 years after SMILE or FS-LASIK, and the change was significantly less pronounced after SMILE.

## Introduction

Corneal transparency is a crucial property of a healthy cornea and can be determined by measuring corneal light backscatter. A technique such as Scheimpflug photography provides objective and quantitative measures of corneal clarity in the form of the corneal densitometry (CD) value. Changes in the cornea, such as edema or inflammation, can lead to opacification and subsequently, an increase in the CD value. Thus, the CD is a useful indicator of early changes in corneal pathologies and can be used to monitor postsurgical stromal reactions, keratocyte activation, or haze. It is also a good predictor of visual acuity (1) and visual quality (2).

Femtosecond laser small incision lenticule extraction (SMILE) is a new technique to correct myopia and myopic astigmatism and is thought to change corneal transparency. CD value is significantly increased during the early postoperative period, as compared to the preoperative period (3–7), and gradually returned to baseline (8–10). This may partly explain the transient haze-like reaction and slower visual recovery after SMILE. Han et al. (4) reported that the CD value was significantly lower than baseline after 3 years; however, the authors did not provide a detailed explanation. Studies to follow CD for a longer time postoperatively are needed to clarify the effect of surgery in terms of persistent corneal transparency in the long term.

This study aimed to objectively assess the changes in CD after SMILE vs. femtosecond laser-assisted LASIK (FS-LASIK) and examine the relationship between other clinical characteristics, such as the type of surgery, patient age, preoperative spherical equivalent (SE), central corneal thickness (CCT), and lenticule thickness (LT)/ablation depth (AD). To the best of our knowledge, this is the first report of a 5-year outcome of SMILE and FS-LASIK for corneal transparency using the densitometry module of the Pentacam Scheimpflug imaging system.

## Materials and Methods

### Patients and Follow-Up

In this prospective study, 67 subjects with myopia and myopic astigmatism who underwent refractive surgery of SMILE (31 left and 35 right eyes of 38 patients) or FS-LASIK (28 left and 26 right eyes of 29 patients) at Fudan University Eye and ENT Hospital (Shanghai, China) from December 2011 to January 2013 were recruited. Inclusion criteria were as follows: (1) age ≥ 18 years; (2) stable refractive error with minimum calculated residual corneal stromal bed thickness of ≥280 μm; (3) preoperative spherical error up to −10.0 D and cylinder up to −5.0 D. Patients with other preexisting ocular diseases were excluded.

The study was approved by the Ethical Committee of Fudan University EENT Hospital Review Board, and conducted in accordance with the tenets of the Declaration of Helsinki. In all patients, a detailed explanation of the risks and benefits of the study was provided and signed informed consent was obtained. Indication for surgery was according to the surgeon's clinical judgment and implemented after careful deliberation of choice of the procedure by patients under the surgeon's guidance.

In all patients, an ocular examination was conducted preoperatively and postoperatively. The main assessments were as follows: (1) Axial length (Zeiss Humphrey IOL Master), intraocular pressure, and slit-lamp evaluation of the anterior segment; (2) visual acuity, including uncorrected distance visual acuity (UDVA), corrected distance visual acuity (CDVA), and objective, manifest and cycloplegic refraction; (3) corneal topography (Pentacam HR, version 1.21r43, Type 70900; Oculus Optikgeräte GmbH, Wetzlar, Germany). The Pentacam scans were taken in automatic release mode, and the CCT, CD values, and wavefront aberrations of the anterior corneal surface over a 6.0 mm central diameter were collected from a single measurement at each visit. All the preoperative measurement was done by the same technician (LW), and all the follow-up measurement was done by the same ophthalmologist (MYL).

Patients were followed for 5 years.

### Surgical Technique

SMILE was performed using a 500 kHz VisuMax femtosecond laser system (Carl Zeiss Meditec AG, Jena, Germany) in expert mode at the following settings for all the eyes: pulse energy of 130 nJ, corneal cap thickness of 100 μm, and optical zone diameter of 6.2–6.8 mm (depending on the preoperative corneal thickness and the refractive error to be corrected). The side cuts were set to 90° (12-o'clock position) with 2.0-mm circumferential width at the superior position. The technical procedure was conducted as previously described (11).

FS-LASIK was performed using a 500 kHz VisuMax femtosecond laser (Carl Zeiss Meditec AG, Jena, Germany) for flap creation and a 250 Hz MEL-80 excimer laser (Carl Zeiss Meditec AG, Jena, Germany) for stromal ablation at the following settings for all the eyes: pulse energy of 185 nJ, flap diameter of 8.0 mm, flap thickness of 90 μm, hinge length of 4.0 mm, and optical zone diameter of 6.2–6.8 mm. In all patients, bandage soft contact lenses (ACUVE OASYS, Inc., FL, USA) were applied for 1 day after surgery.

All surgeries were performed by the same skilled surgeon in both procedures (XZ).

Both groups received the same postoperative topical medications: levofloxacin 4 times per day for 7 days; 0.1% fluorometholone 8 times per day tapered to 1 time per day over 24 days, and artificial tears 4 times per day for 1 month.

### Corneal Densitometry Analysis

**Figure 1:**
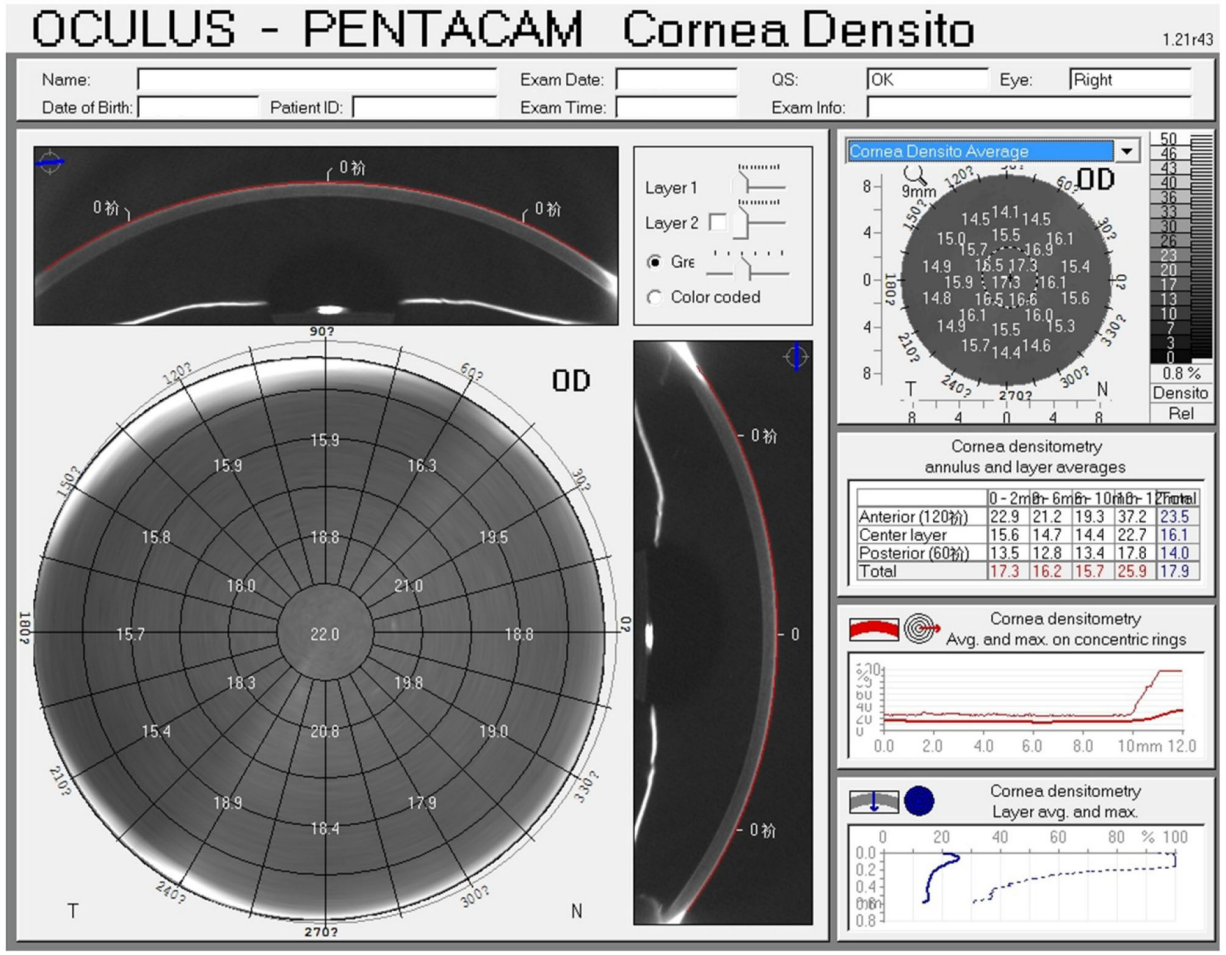
Corneal densitometry map using the Pentacam Scheimpflug imaging system.

CD was quantified with the Pentacam HR (software version 1.21r43) (Figure 1). The device captures 25 images from 0° to 180° and automatically records 12,500 elevation points in 2 s, and simultaneously collects corneal backscattered light in the different regions of the cornea and transforms it into CD; the CD values are recorded in standardized grayscale units (GSU) at a range from 0 (maximum transparency) to 100 (minimum transparency). The data are displayed on the CD map, which is divided into three anatomical layers based on the depth: The anterior layer (120 μm anteriorly), posterior layer (60 μm posteriorly), and central layer (at mid-distance between the two layers). In addition, four concentric radial zones are defined around the corneal apex (0–2, 2–6, 6–10, and 10–12 mm). In patients with white to white (WTW) diameter of <1 mm, a part of the limbus and sclera may be included in the measurement (12); hence, values at the outermost zone of 10–12 mm have the weakest reliability and reproducibility and were excluded. An annulus measuring 0–6 mm is considered to be in the optic zone and is most likely to affect visual outcomes.

### Data and Statistical Analysis

Data were analyzed using R 3.5.3 (R Project for Statistical Computing, http://cran.r-project.org). Continuous variables are presented as mean ± standard deviation (SD), and categorical variables, as frequency. For comparison studies between the SMILE and FS-LASIK groups, two-sample *t*-test and Chi-square test, respectively were used for the age and sex at baseline, and linear mixed models were constructed for the other baseline variables and changes of CD; the latter method was also used to compare the change of CD with time in each group and assess the relationship between the change of CD and type of surgery, age, SE, CCT, and LT/AD. In linear mixed models, subject ID was included as the random intercept to account for the relationship between right and left eyes in one subject, and other variables of interest were included as fixed effects. A *P*-value of < 0.05 was considered to be statistically significant.

## Results

In total, 67 patients with myopia and myopic astigmatism who underwent SMILE (31 left and 35 right eyes of 38 patients) or FS-LASIK (28 left and 26 right eyes of 29 patients) were included in the prospective study. The preoperative baseline characteristics and CD values are shown in Tables 1, 2. In total, 89.6% of patients (33 SMILE patients and 27 FS-LASIK patients) were available for the full 5-year duration of postoperative follow-up. No complications or adverse events were observed.

**Table 1:** Patient profiles.

**Characteristics**	**SMILE group (*n* = 66 eyes)**	**FS-LASIK group (*n* = 54 eyes)**	*P* value^*^
Age (mean ± SD, year)	29.2 ± 5.5	28.8 ± 7.0	0.78
Range of age (year)	22, 44	19, 47	
Sex (male/female)	13/25	5/24	0.20
Axial length (mm)	26.33 ± 0.91	26.16 ± 1.04	0.55
Range of axial length (μm)	24.28, 28.22	24.51, 29.97	
Preoperative spherical error (D)	−6.48 ± 1.25	−6.95 ± 1.57	0.11
Range (D)	−9.25, −3.50	−9.50, −3.00	
Preoperative cylinder (D)	−0.89 ± 0.72	−1.07 ± 0.74	0.25
Range (D)	−3.00, 0	−3.25, 0	
Preoperative SE (D)	−6.98 ± 1.07	−7.48 ± 1.62	0.09
Range (D)	−9.25, −5.00	−10.00, −3.38	
Preoperative CCT (μm)	552.9 ± 28.5	545.9 ± 24.1	0.27
Range (μm)	502, 614	504, 592	
LT/AD (μm)	136.4 ± 17.7	134.7 ± 22.9	0.89
Range (μm)	92, 170	72, 168	
CDVA (logMAR)	−0.03 ± 0.05	−0.01 ± 0.04	0.26
Range (logMAR)	−0.18, 0.10	−0.08, 0.10	

* *Continuous variables were analyzed by t-test for subject-level variables or linear mixed model for eye-level variables. Frequency data were analyzed by Chi-squared test; P < 0.05*.

*LT, lenticule thickness for SMILE patients; AD, ablation depth for FS-LASIK patients; CCT, central corneal thickness; UDVA, uncorrected distance visual acuity; CDVA, corrected distance visual acuity; SE, spherical equivalent*.

**Table 2:** Corneal densitometry (CD) after SMILE and FS-LASIK.

**CD (GSU)**		**Anterior layer**			**Central layer**			**Posterior layer**			**Total thickness**		
		**0–2 mm**	**2–6 mm**	**6–10 mm**	**0–2 mm**	**2–6 mm**	**6–10 mm**	**0–2 mm**	**2–6 mm**	**6–10 mm**	**0–2 mm**	**2–6 mm**	**6–10 mm**
Pre-op	SMILE	25.24 ± 3.83	23.29 ± 3.48	23.49 ± 4.48	16.87 ± 2.13	15.49 ± 1.90	16.22 ± 2.62	14.46 ± 2.07	13.67 ± 1.89	15.21 ± 2.39	18.87 ± 2.61	17.49 ± 2.37	18.30 ± 3.07
	FS-LASIK	27.26 ± 3.26	24.90 ± 2.80	24.22 ± 2.72	18.04 ± 1.80	16.43 ± 1.51	16.76 ± 1.43	15.30 ± 1.85	14.49 ± 1.60	16.16 ± 1.48	20.19 ± 2.22	18.60 ± 1.89	19.04 ± 1.71
	*P*^§^	0.051	0.09	0.50	0.04^||^	0.06	0.42	0.13	0.12	0.12	0.06	0.08	0.33
Post-op month 6–12	SMILE	25.85 ± 4.93	24.12 ± 4.40	23.08 ± 4.65	17.09 ± 2.78	15.71 ± 2.28	16.18 ± 2.83	14.36 ± 2.65	13.95 ± 2.35	15.70 ± 3.06	19.11 ± 3.42	17.93 ± 2.98	18.32 ± 3.45
	Change	0.33 ± 4.31	0.57 ± 3.81	−0.91 ± 3.52	−0.02 ± 2.30	−0.01 ± 1.89	−0.40 ± 1.82	−0.36 ± 2.37	0.04 ± 2.10	0.20 ± 2.12	−0.02 ± 2.94	0.19 ± 2.58	−0.36 ± 2.41
	FS-LASIK	26.23 ± 2.87	24.61 ± 2.80	22.97 ± 2.87	16.82 ± 1.79	15.81 ± 1.59	16.19 ± 1.62	13.99 ± 1.73	14.00 ± 1.47	15.96 ± 1.43	19.02 ± 1.98	18.15 ± 1.84	18.38 ± 1.82
	Change	−1.40 ± 4.39	−0.50 ± 3.77	−1.83 ± 3.73^#^	−1.37 ± 2.48^#^	−0.74 ± 2.02	−0.92 ± 2.09^#^	−1.47 ± 2.52^#^	−0.53 ± 2.13	−0.16 ± 2.03	−1.39 ± 3.03^#^	−0.59 ± 2.56	−0.97 ± 2.48^#^
	^Δ^Change^§^	−1.01	−0.48	−0.58	−0.99	−0.45	−0.35	−0.76	−0.26	−0.12	−0.90	−0.39	−0.36
	95% CI^§^	(−4.32, 2.30)	(−3.36, 2.39)	(−3.04, 1.88)	(−2.75, 0.76)	(−1.86, 0.96)	(−1.63, 0.93)	(−2.57, 1.04)	(−1.83, 1.31)	(−1.58, 1.33)	(−3.14, 1.35)	(−2.32, 1.54)	(−2.02, 1.30)
Post-op year 5	SMILE	18.95 ± 5.10	17.69 ± 4.81	18.26 ± 5.48	12.28 ± 3.30	11.40 ± 2.95	12.60 ± 3.42	9.92 ± 2.78	9.77 ± 2.60	11.55 ± 2.90	13.71 ± 3.56	12.96 ± 3.34	14.13 ± 3.85
	Change	−5.85 ± 5.13^*^	−5.17 ± 4.76^*^	−5.22 ± 4.66^*^	−4.32 ± 3.11^*^	−3.84 ± 2.79^*^	−3.60 ± 2.70^*^	−4.38 ± 3.36^*^	−3.74 ± 2.99^*^	−3.57 ± 2.65^*^	−4.86 ± 3.66^*^	−4.25 ± 3.37^*^	−4.12 ± 3.21^*^
	FS-LASIK	18.49 ± 5.43	17.18 ± 4.89	16.82 ± 4.38	11.86 ± 3.49	11.13 ± 3.11	11.82 ± 2.99	9.80 ± 3.06	9.64 ± 2.78	11.18 ± 2.79	13.38 ± 3.83	12.65 ± 3.45	13.28 ± 3.27
	Change	−8.87 ± 5.61^#^	−7.79 ± 5.09^#^	−7.44 ± 4.72^#^	−6.22 ± 3.72^#^	−5.33 ± 3.35^#^	−4.94 ± 3.11^#^	−5.52 ± 3.54^#^	−4.86 ± 3.24^#^	−4.99 ± 3.04^#^	−6.87 ± 4.15^#^	−5.98 ± 3.77^#^	−5.78 ± 3.51^#^
	^Δ^Change^§^	−3.04^†^	−2.66^†^	−2.25^†^	−1.90^†^	−1.49^†^	−1.35^†^	−1.14	−1.10	−1.41^†^	−2.01^†^	−1.74^†^	−1.66^†^
	95% CI^§^	(−5.35, −0.73)	(−4.83, −0.49)	(−4.40, −0.10)	(−3.25, −0.55)	(−2.73, −0.26)	(−2.57, −0.12)	(−2.74, 0.46)	(−2.51, 0.30)	(−2.63, −0.19)	(−3.61, −0.42)	(−3.20, −0.28)	(−3.10, −0.23)

|| *P < 0.05, significantly different in preoperative values between SMILE and FS-LASIK group*.

* *P < 0.05, significantly different from the preoperative values in the SMILE group*.

# *P < 0.05, significantly different from the preoperative values in the FS-LASIK group*.

† *P < 0.05, a greater change in FS-LASIK group than SMILE group*.

§ *Estimated from the linear mixed model with subject ID as the random intercept*.

Δ *Change = difference in the changes between the two groups; CI = confidence interval; GSU = grayscale units*.

### Postoperative Refraction

At 5-year follow-up, 83.9% (47/56) of SMILE-treated eyes and 89.6% (43/48) FS-LASIK-treated eyes had a postoperative logMAR UDVA of 0 or better. The mean spherical error is −0.03 ± 0.49 for SMILE and −0.29 ± 0.62 for FS-LASIK. The mean cylinder is −0.26 ± 0.24 for SMILE and −0.28 ± 0.28 for FS-LASIK. There were no significant differences between SMILE and FS-LASIK for spherical error and cylinder.

### Postoperative Outcomes on Corneal Densitometry

**Figure 2:**
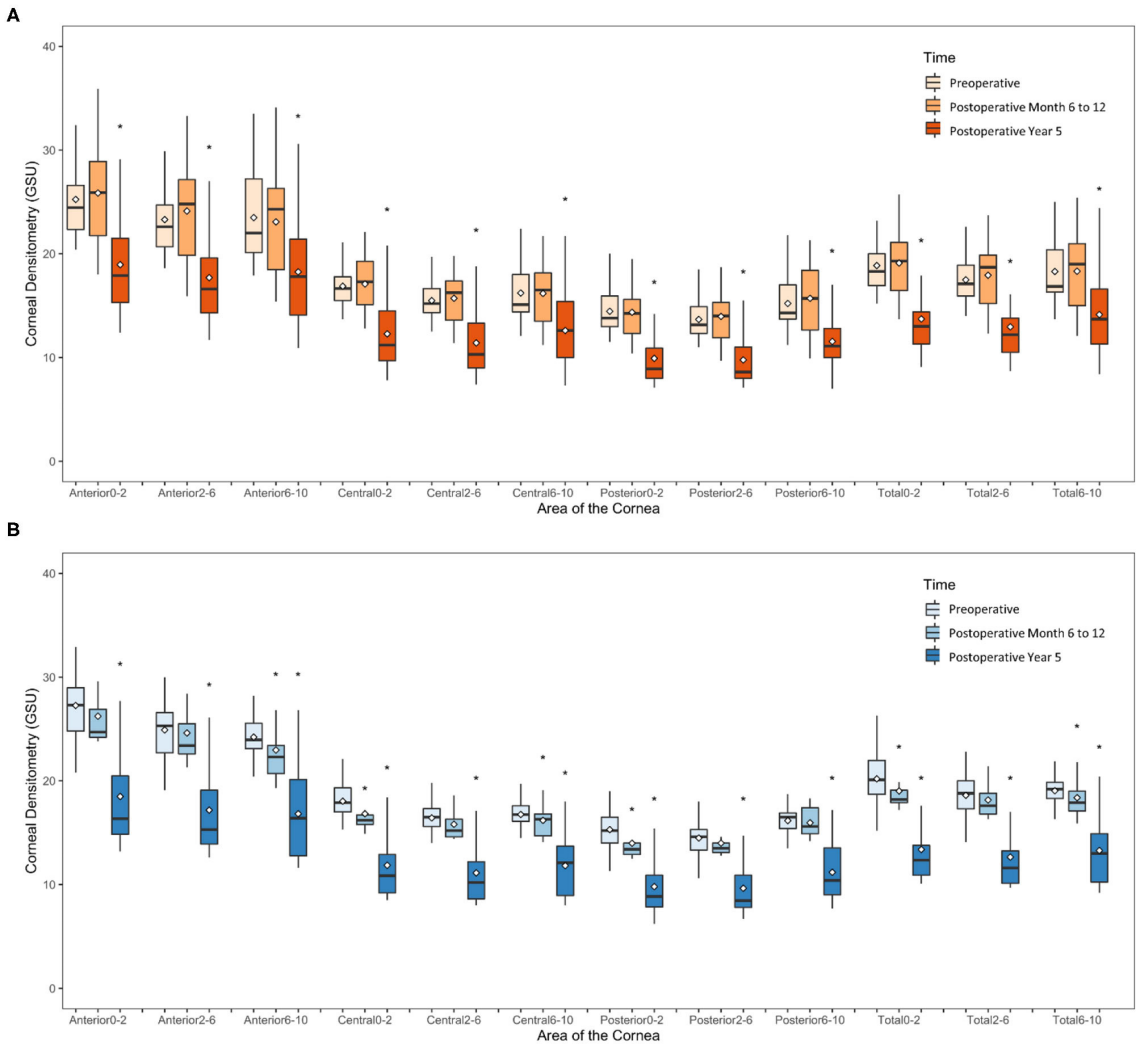
Time course of corneal densitometry in the SMILE and FS-LASIK group. **(A)** SMILE group; **(B)** FS-LASIK group. *p < 0.05, significantly different from the preoperative values.

**Figure 3:**
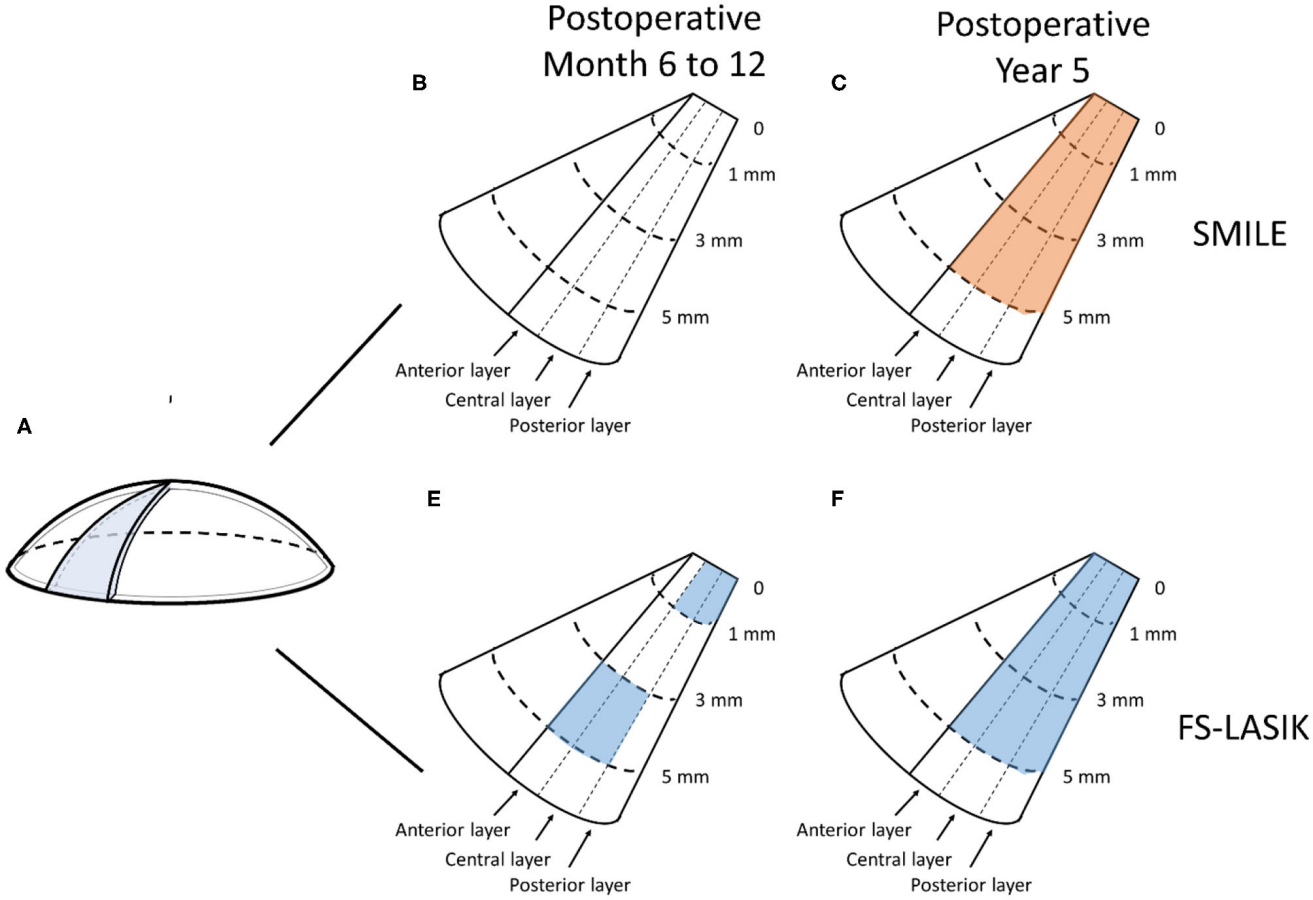
Changes of corneal densitometry (CD) at 6–12 months and 5 years after SMILE and FS-LASIK. **(A)** Diagram of the cornea; **(B–E)** Changes of CD relative to baseline (blue for SMILE and red for FS-LASIK presents a statistically significant reduction); **(B)** SMILE at 6–12 months postoperatively; **(C)** SMILE at 5 years postoperatively; **(D)** FS-LASIK at 6–12 months postoperatively; **(E)** FS-LASIK at 5 years postoperatively.

In the SMILE group, the postoperative CD at 6–12 months was similar to the preoperative values (*P* > 0.05); however, the CD values at each of three annuli (0–2, 2–6, and 6–10 mm) of all three layers were significantly decreased at 5 years postoperatively as compared with those at baseline (all *P* < 0.001) (Figures 2A, 3A–C, Table 2).

In the FS-LASIK group, the CD values at the 6–10-mm zone (*P* = 0.007) of the anterior layer, 0–2-mm (*P* = 0.012) and 6–10-mm zones (*P* = 0.031) of the central layer, 0–2-mm zone (*P* = 0.02) of the posterior layer, and 0–2 mm (*P* = 0.026) and 6–10-mm zones (*P* = 0.041) of the total thickness were significantly reduced at 6–12 months postoperatively. The CD values at each of the three annuli of all three layers at postoperative 5 years were significantly reduced (all *P* < 0.001) (Figures 2B, 3D,E, Table 2).

In comparing the changes in CD between the SMILE and FS-LASIK groups, there were no significant differences in the values obtained at each of the annuli at the postoperative timepoint of 6–12 months (*P* > 0.05). However, at the postoperative timepoint of 5 years, the reduction was more apparent in the FS-LASIK group at the anterior layer (*P* = 0.011 for 0–2 mm; *P* = 0.017 for 2–6 mm; *P* = 0.041 for the 6–10-mm zone of the annulus), the central layer (*P* = 0.007 for 0–2 mm; *P* = 0.018 for 2–6 mm; *P* = 0.032 for the 6–10-mm zone of the annulus), and total thickness (*P* = 0.014 for 0–2 mm; *P* = 0.021 for 2–6 mm; *P* = 0.024 for the 6–10-mm zone of the annulus). Similarly, a greater reduction in CD was observed in the FS-LASIK group compared to the SMILE group at the 6–10-mm zone of the posterior layer (*P* = 0.025) (Table 2).

### Factors Associated With Corneal Densitometry After Refractive Surgery

At a postoperative timepoint of 5 years, all CD values except those at the 0–2-mm and 2–6-mm zones of the posterior corneal layers, were significantly associated with the type of surgery (*P* < 0.05) (Table 3). At an earlier postoperative timepoint of 6–12 months, the CD value was not associated with the type of surgery. Moreover, the CD value was not statistically correlated with the other characteristics such as age, SE, CCT, and LT/AD (Table 3).

**Table 3:** Univariate analysis between the change of corneal densitometry after refractive surgery and type of operation, age, SE, CCT, and planned ablation depth/lenticule thickness.

**Variable**	**Postoperative month 6–12**										**Postoperative year 5**									
	**Group^†^**		**Age**		**SE**		**CCT**		**LT/AD**		**Group^†^**		**Age**		**SE**		**CCT**		**LT/AD**	
	**Coef**	***P***	**Coef**	***P***	**Coef**	***P***	**Coef**	***P***	**Coef**	***P***	**Coef**	***P***	**Coef**	***P***	**Coef**	***P***	**Coef**	***P***	**Coef**	***P***
**ANTERIOR LAYER**																				
0–2 mm	−1.012	0.54	0.048	0.74	0.263	0.77	0.011	0.61	−0.026	0.28	−3.043	0.01^*^	0.022	0.83	1.785	0.14	0.007	0.78	0.023	0.50
2–6 mm	−0.481	0.74	0.038	0.76	0.643	0.50	0.012	0.56	−0.025	0.30	−2.659	0.02^*^	0.008	0.93	1.753	0.11	0.007	0.74	0.021	0.50
6–10 mm	−0.58	0.64	−0.002	0.98	0.256	0.84	0.012	0.53	−0.035	0.20	−2.252	0.04^*^	0.021	0.81	0.98	0.34	0.022	0.31	0.004	0.89
**CENTRAL LAYER**																				
0–2 mm	−0.995	0.26	0.028	0.71	−0.037	0.94	0.004	0.75	−0.013	0.36	−1.903	0.007^*^	−0.011	0.85	1.196	0.11	0.006	0.67	0.026	0.18
2–6 mm	−0.449	0.52	0.034	0.58	0.109	0.82	0.005	0.60	−0.01	0.48	−1.494	0.02^*^	−0.007	0.89	0.937	0.16	0.005	0.71	0.026	0.14
6–10 mm	−0.35	0.59	−0.002	0.98	−0.182	0.77	0.006	0.55	−0.011	0.45	−1.347	0.03^*^	−0.002	0.97	0.774	0.22	0.01	0.40	0.02	0.25
**POSTERIOR LAYER**																				
0–2 mm	−0.764	0.40	0.012	0.88	−0.182	0.71	0.004	0.77	−0.016	0.31	−1.138	0.16	0.001	0.99	0.802	0.28	0.02	0.21	0	1.00
2–6 mm	−0.255	0.74	0.024	0.72	0.161	0.72	0.005	0.68	−0.013	0.36	−1.102	0.12	−0.005	0.93	0.814	0.23	0.015	0.27	0.008	0.65
6–10 mm	−0.124	0.86	0.042	0.50	−0.028	0.97	0.004	0.75	−0.011	0.51	−1.408	0.03^*^	−0.001	0.98	0.9	0.15	0.013	0.29	0.016	0.36
**TOTAL THICKNESS**																				
0–2 mm	−0.899	0.42	0.032	0.74	0.01	0.99	0.006	0.69	−0.019	0.29	−2.013	0.01^*^	0.007	0.92	1.291	0.13	0.009	0.57	0.015	0.52
2–6 mm	−0.391	0.68	0.031	0.71	0.293	0.64	0.007	0.62	−0.017	0.34	−1.737	0.02^*^	−0.001	0.98	1.18	0.13	0.008	0.61	0.018	0.40
6–10 mm	−0.361	0.66	0.014	0.85	0.001	0.99	0.007	0.58	−0.019	0.33	−1.664	0.02^*^	0.007	0.91	0.898	0.22	0.014	0.33	0.012	0.55

* *P < 0.05*.

† *SMILE, 0; FS-LASIK, 1*.

*CCT, central corneal thickness; SE, spherical equivalent; LT, lenticule thickness; AD, ablation depth; Coef, univariate coefficient*.

### Relationships Between Corneal Densitometry and Corneal Wavefront Aberrations

There was no difference in preoperative wavefront aberrations of the cornea between the SMILE and FS-LASIK groups. However, at 5 years after surgery, anterior corneal higher-order aberrations (HOA) were significantly higher in the SMILE group as opposed to FS-LASIK. In particular, the Z(3, −1), coma, spherical aberration, third-order aberration, and total HOA were higher (Supplementary Table 1). There was no detectable relationship between changes in CD and changes in corneal wavefront aberrations (Supplementary Table 2).

## Discussion

Characterizing the long-term changes to the corneal structure after SMILE is critical for optimizing surgical outcomes. CD is an objective measurement to assess corneal transparency. It is increasingly being used to routinely monitor postsurgical outcomes after corneal collagen crosslinking (13), keratoplasty (1, 14), and corneal refractive surgery (15). However, the investigations regarding postoperative changes in CD after SMILE and FS-LASIK are relatively fewer and with inconsistent results (Supplementary Table 3). To the best of our knowledge, our study is the first 5-year prospective study that compares CD values after two types of refractive surgeries, SMILE and FS-LASIK, with Scheimpflug imaging.

In the present study, CD at all examined zones decreased significantly at 5 years postoperatively compared with baseline in both SMILE and FS-LASIK groups. Previous literature has shown that CD was decreased at 3 months (16) and 1 year (17) after photorefractive keratectomy (PRK). Lazaridis et al. (8) and Han et al. (4) compared the changes in CD after SMILE and FS-LASIK and reported a significant decrease at 3 months and 3 years after either procedure. The present study further explores the long-term changes in corneal transparency after these two procedures. The decrease in CD could be a result of reduced backscatter constituents, since the amount, size, and organization of keratocytes and stromal collagen fibrils have an impact on the optical properties of the cornea (18). Lazaridis et al. (8) reported a weak negative association between the CD in the 0–6-mm zone 3 months after SMILE/FS-LASIK and the amount of the extracted or ablated stromal tissue. Besides, a decrease of the keratocyte density after SMILE/FS-LASIK with *in vivo* confocal microscopy (IVCM) was observed (19), which may increase the corneal clarity. Decreased corneal hydration after laser refractive surgery reported by Patel et al. (20) could also lead to the change of CD. Admittedly, the manual operation of different surgeons could affect the postoperative recovery and thus, the change of CD values.

The decrease of CD in most zones was of no significance at 6–12 months after SMILE and FS-LASIK but significant at 5 years postoperatively, which indicated that corneal transparency continues to improve over a long time. To explain the dynamic change in CD, one possible contributing factor is the corneal wound healing response. We found that bright particles at the interface under the cap/flap diminished with time but were still present at 5 years postoperatively (manuscript submitted for publication). These small particles were thought to be transient particles such as the ocular surface debris (lipid products), stress fiber bundles in migratory keratocytes, or permanent interface particles derived from surgical instruments (21), and may affect the optical properties of the cornea.

Our analysis revealed that type of surgery, rather than age, SE, or LT/AD, was associated with changes in CD values. We also observed significantly smaller changes in CD in the anterior (except 6–10 mm) and central layer at long-term in SMILE vs. FS-LASIK, which is in agreement with the findings of a 3-month study (8). In a SMILE procedure, there may be a misalignment of the cap and stroma interface (tent effect) after lenticule extraction. This can cause structural changes near the incision site and twisting of the Bowman's layer (microdistortions), which remain up to 3 years after SMILE (22). These subtle structural changes may have a long-standing impact on corneal optical properties and increase the cornea backscatter. Besides the surgical approach, different laser mechanisms may also lead to the differences in CD. During the SMILE, the femtosecond laser photodisruption is mainly applied, while in FS-LASIK, the cornea is burdened with additional excimer laser photoablation. The distinction may cause a different postoperative healing response. In animal models, less tissue inflammation, keratocyte response, and early extracellular matrix deposition was observed in the rabbit cornea at early postoperative periods after SMILE than compared to FS-LASIK (23), indicative of the differences in repair patterns of the stroma after either procedure. Further studies are needed to clarify whether it can cause the changes of CD observed. Since the sample size was relatively small, caution should be applied, as the findings might be caused by the individual patient differences. Though reports indicate that CD was associated with age (24, 25) and LT/AD (8), such relationships were not detected here.

The reduced CD may affect visual quality after refractive surgery though no relationship between changes in CD and corneal wavefront aberrations was found here. There are few studies regarding the relationship between CD and aberrations after refractive surgery, but the correlations were reported in children with vernal keratoconjunctivitis (26) and patients after penetrating keratoplasty (27). One reason we did not detect it could be that the effect of refractive surgery on the residual cornea is uneven and the mean CD of each layer divided by the Scheimpflug imaging system cannot fully represent the change of corneal organization after surgery. The contrast sensitivity may also be affected. Additionally, change in CD may impact the subjective quality of vision and be related to symptoms such as glare and halos. More-refined research could be further undertaken to investigate the clinical correlations of postoperative CD.

This study has some limitations. First, the sample size was relatively small, and we were unable to determine the association between the changes of CD and the other clinical characteristics. Further studies with larger sample sizes are needed. Second, CD values were obtained with Scheimpflug analysis which is a non-contact method. This may cause greater reflection at the interfaces between the layers and make it difficult to distinguish between backscattered and reflected light (28, 29); IVCM analysis can overcome this disadvantage. Studies that use IVCM-generated densitometry to further characterize the wound-healing response after SMILE are needed to validate the current results and elucidate the mechanism of the decrease of CD.

In summary, the present study found that the CD value as measured by Scheimpflug imaging was significantly decreased at 5 years after SMILE or FS-LASIK refractive surgery. Postoperative changes in CD were smaller in the SMILE group at long-term follow-up compared to FS-LASIK. Further research can be undertaken to investigate the association between postoperative CD and visual quality. Studies to evaluate the long-term cellular and molecular changes in the cornea after corneal refractive surgery are needed to clarify the mechanism of reduction of CD and optimize postoperative management.

## Data Availability Statement

The datasets generated for this study are available on request to the corresponding author.

## Ethics Statement

The studies involving human participants were reviewed and approved by Ethics Committee of Fudan University Eye and ENT Hospital Review Board (Shanghai, China). The patients/participants provided their written informed consent to participate in this study.

## Author Contributions

XZ: study concept and design. WY, RW, DF, YZ, and JS: data collection. ML, JZ, RW, and YS: analysis and interpretation of data. RW: writing the manuscript. ML, WY, YS, and JC: critical revision of the manuscript. XZ: supervision. All authors read and approved the final manuscript.

## Conflict of Interest

The authors declare that the research was conducted in the absence of any commercial or financial relationships that could be construed as a potential conflict of interest.

## References

[1] Schaub F Gerber F Adler W Enders P Schrittenlocher S Heindl LM. Corneal densitometry as a predictive diagnostic tool for visual acuity results after descemet membrane endothelial keratoplasty. Am. J. Ophthalmol. (2019) 198:124–9. 10.1016/j.ajo.2018.10.002 30315754

[2] Patel SV McLaren JW Hodge DO Bourne WM. The effect of corneal light scatter on vision after penetrating keratoplasty. Am. J. Ophthalmol. (2008) 146:913–9. 10.1016/j.ajo.2008.07.018 18774549 PMC2612528

[3] Agca A Ozgurhan EB Yildirim Y Cankaya KI Guleryuz NB Alkin Z. Corneal backscatter analysis by *in vivo* confocal microscopy: fellow eye comparison of small incision lenticule extraction and femtosecond laser-assisted LASIK. J. Ophthalmol. (2014) 2014:265012. 10.1155/2014/265012 24734168 PMC3964686

[4] Han T Zhao J Shen Y Chen Y Tian M Zhou X. A three-year observation of corneal backscatter after small incision lenticule extraction (SMILE). J. Refract. Surg. (2017) 33:377–82. 10.3928/1081597X-20170420-01 28586497

[5] Liu T Dan T Luo Y. Small incision lenticule extraction for correction of myopia and myopic astigmatism: first 24-hour outcomes. J. Ophthalmol. (2017) 2017:5824534. 10.1155/2017/5824534 28680704 PMC5478873

[6] Patel SV Maguire LJ McLaren JW Hodge DO Bourne WM. Femtosecond laser versus mechanical microkeratome for LASIK: a randomized controlled study. Ophthalmology. (2007) 114:1482–90. 10.1016/j.ophtha.2006.10.057 17350688

[7] Savini G Huang J Lombardo M Serrao S Schiano-Lomoriello D Venanzio S. Objective monitoring of corneal backward light scattering after femtosecond laser-assisted LASIK. J. Refract. Surg. (2016) 32:20–5. 10.3928/1081597X-20151207-08 26812710

[8] Lazaridis A Droutsas K Sekundo W Petrak M Schulze S. Corneal clarity and visual outcomes after small-incision lenticule extraction and comparison to femtosecond laser-assisted *in situ* keratomileusis. J. Ophthalmol. (2017) 2017:5646390. 10.1155/2017/5646390 28396803 PMC5370519

[9] Pedersen IB Ivarsen A Hjortdal J. Changes in astigmatism, densitometry, and aberrations after SMILE for low to high myopic astigmatism: a 12-month prospective study. J. Refract. Surg. (2017) 33:11–7. 10.3928/1081597X-20161006-04 28068441

[10] Shajari M Wanner E Rusev V Mir Mohi Sefat S Mayer WJ Kohnen T. Corneal densitometry after femtosecond laser-assisted in situ keratomileusis (Fs-LASIK) and small incision lenticule extraction (SMILE). Curr. Eye Res. (2018) 43:605–10. 10.1080/02713683.2018.1431288 29537886

[11] Li M Zhao J Miao H Shen Y Sun L Tian M. Mild decentration measured by a Scheimpflug camera and its impact on visual quality following SMILE in the early learning curve. Invest. Ophthalmol. Vis. Sci. (2014) 55:3886–92. 10.1167/iovs.13-13714 24845636

[12] Cakmak HB Cagil N Simavli H Raza S. Corneal white-to-white distance and mesopic pupil diameter. Int. J. Ophthalmol. (2012) 5:505–9. 22937514 10.3980/j.issn.2222-3959.2012.04.19 PMC3428550

[13] Alnawaiseh M Rosentreter A Eveslage M Eter N Zumhagen L. Changes in corneal transparency after cross-linking for progressive keratoconus: long-term follow-up. J. Refract. Surg. (2015) 31:614–8. 10.3928/1081597X-20150820-07 26352567

[14] Alnawaiseh M Rosentreter A Prokosch V Eveslage M Eter N Zumhagen L. Changes in corneal densitometry in patients with fuchs endothelial dystrophy after endothelial keratoplasty. Curr. Eye Res. (2017) 42:163–7. 10.3109/02713683.2016.1146774 27260144

[15] Takacs AI Mihaltz K Nagy ZZ. Corneal density with the pentacam after photorefractive keratectomy. J. Refract. Surg. (2011) 27:269–77. 10.3928/1081597X-20100618-02 20672772

[16] Boulze-Pankert M Dariel R Hoffart L. Corneal scheimpflug densitometry following photorefractive keratectomy in myopic eyes. J. Refract. Surg. (2016) 32:788–91. 10.3928/1081597X-20160720-02 27824385

[17] Cennamo G Forte R Aufiero B La Rana A. Computerized scheimpflug densitometry as a measure of corneal optical density after excimer laser refractive surgery in myopic eyes. J. Cataract Refract. Surg. (2011) 37:1502–6. 10.1016/j.jcrs.2011.03.037 21782093

[18] Boote C Dennis S Newton RH Puri H Meek KM. Collagen fibrils appear more closely packed in the prepupillary cornea: optical and biomechanical implications. Invest. Ophthalmol. Vis. Sci. (2003) 44:2941–8. 10.1167/iovs.03-0131 12824235

[19] Li M Niu L Qin B Zhou Z Ni K Le Q. Confocal comparison of corneal reinnervation after small incision lenticule extraction (SMILE) and femtosecond laser in situ keratomileusis (FS-LASIK). PLoS ONE. (2013) 8:e81435. 10.1371/journal.pone.0081435 24349069 PMC3857190

[20] Patel S., Alió, J. L., Javaloy, J., Perez-Santonja, J. J., Artola, A., and Rodriguez-Prats, J. (2008). Human cornea before and after refractive surgery using a new device: VCH-1. Cornea. 27, 1042–1049. 10.1097/ICO.0b013e318172fc40 18812769

[21] Sonigo B. Iordanidou V. Chong-Sit D. Auclin F. Ancel J. M. Labbé A.. (2006). *In vivo* corneal confocal microscopy comparison of intralase femtosecond laser and mechanical microkeratome for laser *in situ* keratomileusis. Invest. Ophthalmol. Vis. Sci. 47, 2803–11. 10.1167/iovs.05-1207 16799017

[22] Zhao J Gao Y Han T Zeng L Miao H Yang D. Microdistortions in Bowman's layer 3 years after SMILE for myopia. J. Refract. Surg. (2019) 35:96–101. 10.3928/1081597X-20181212-01 30742223

[23] Dong Z Zhou X Wu J Zhang Z Li T Zhou Z. Small incision lenticule extraction (SMILE) and femtosecond laser LASIK: comparison of corneal wound healing and inflammation. Br. J. Ophthalmol. (2014) 98:263–9. 10.1136/bjophthalmol-2013-303415 24227802 PMC3913294

[24] Ní Dhubhghaill S Rozema JJ Jongenelen S Ruiz Hidalgo I Zakaria N Tassignon M-J. Normative values for corneal densitometry analysis by scheimpflug optical assessment. Invest. Opthalmol. Vis. Sci. (2014) 55:162. 10.1167/iovs.13-13236 24327608

[25] Garzón N Poyales F Illarramendi I Mendicute J Jáñez Ó Caro P. Corneal densitometry and its correlation with age, pachymetry, corneal curvature, and refraction. Int. Ophthalmol. (2017) 37:1263–8. 10.1007/s10792-016-0397-y 27837355

[26] Chan TCY Wong ES Chan JCK Wang Y Yu M Maeda N. Corneal backward scattering and higher-order aberrations in children with vernal keratoconjunctivitis and normal topography. Acta Ophthalmol. (2018) 96:e327–e333. 10.1111/aos.13566 29090512

[27] Kobashi H Kamiya K Shimizu K. Impact of forward and backward scattering and corneal higher-order aberrations on visual acuity after penetrating keratoplasty. Semin. Ophthalmol. (2018) 33:748–56. 10.1080/08820538.2018.1427767 29336641

[28] Lohmann CP Timberlake GT Fitzke FW Gartry DS Muir MK Marshall J. Corneal light scattering after excimer laser photorefractive keratectomy: the objective measurements of haze. Refract. Corneal Surg. (1992) 8:114–21. 1591203

[29] Patel SV Winter EJ McLaren JW Bourne WM. Objective measurement of backscattered light from the anterior and posterior cornea *in vivo*. Invest. Ophthalmol. Vis. Sci. (2007) 48:166–72. 10.1167/iovs.06-0767 17197529

