# Supplementary material for: Corneal Densitometry After Small Incision Lenticule Extraction (SMILE) and Femtosecond Laser-Assisted LASIK (FS-LASIK): 5-Year Prospective Comparative Study
Source: Front Med (Lausanne). 2020 Nov 6;7:521078. doi: 10.3389/fmed.2020.521078 (PMC7681246; doi:10.3389/fmed.2020.521078)
Supplement: Supplementary file 1 [file Data_Sheet_1.docx]

Supplementary Material

# Supplementary Figures and Tables

**Supplementary Table 1.** Comparison of Corneal Wavefront Aberrations between Groups

| Wavefront aberrations | Preoperative | | | Postoperative Year 5 | | |
| --- | --- | --- | --- | --- | --- | --- |
|  | SMILE | FS-LASIK | *P* | SMILE | FS-LASIK | *P* |
| **Front cornea** |  |  |  |  |  |  |
| HOAs | 0.39 ± 0.09 | 0.40 ± 0.10 | 0.40 | 0.94 ± 0.34 | 0.87 ± 0.23 | 0.32 |
| Coma | 0.20 ± 0.11 | 0.19 ± 0.11 | 0.83 | 0.75 ± 0.36 | 0.50 ± 0.28 | 0.002* |
| Z(3,-1) | 0.16 ± 0.12 | 0.15 ± 0.12 | 0.60 | 0.64 ± 0.38 | 0.30 ± 0.24 | <0.001* |
| Z(3,1) | 0.08 ± 0.06 | 0.09 ± 0.07 | 0.39 | 0.31 ± 0.22 | 0.33 ± 0.26 | 0.64 |
| Third-order | 0.22 ± 0.10 | 0.22 ± 0.11 | 0.90 | 0.77 ± 0.36 | 0.54 ± 0.26 | 0.002* |
| Spherical aberration | 0.25 ± 0.08 | 0.27 ± 0.08 | 0.45 | 0.43 ± 0.15 | 0.61 ± 0.16 | <0.001* |
| **Back cornea** |  |  |  |  |  |  |
| HOAs | 0.19 ± 0.03 | 0.19 ± 0.03 | 0.54 | 0.21 ± 0.04 | 0.21 ± 0.03 | 0.54 |
| Coma | 0.04 ± 0.03 | 0.04 ± 0.02 | 0.94 | 0.06 ± 0.03 | 0.05 ± 0.03 | 0.63 |
| Z(3,-1) | 0.03 ± 0.03 | 0.03 ± 0.03 | 0.92 | 0.04 ± 0.04 | 0.04 ± 0.03 | 0.87 |
| Z(3,1) | 0.02 ± 0.02 | 0.02 ± 0.01 | 0.46 | 0.03 ± 0.02 | 0.03 ± 0.02 | 0.90 |
| Third-order | 0.07 ± 0.04 | 0.06 ± 0.03 | 0.35 | 0.10 ± 0.06 | 0.09 ± 0.04 | 0.46 |
| Spherical aberration | 0.16 ± 0.03 | 0.15 ± 0.02 | 0.82 | 0.16 ± 0.03 | 0.17 ± 0.03 | 0.14 |
| **Total cornea** |  |  |  |  |  |  |
| HOAs | 0.38 ± 0.10 | 0.40 ± 0.11 | 0.49 | 1.00 ± 0.36 | 0.90 ± 0.25 | 0.20 |
| Coma | 0.21 ± 0.10 | 0.19 ± 0.12 | 0.64 | 0.81 ± 0.40 | 0.53 ± 0.31 | 0.001* |
| Z(3,-1) | 0.17 ± 0.12 | 0.15 ± 0.12 | 0.50 | 0.68 ± 0.42 | 0.32 ± 0.26 | <0.001* |
| Z(3,1) | 0.08 ± 0.06 | 0.09 ± 0.07 | 0.57 | 0.33 ± 0.24 | 0.35 ± 0.28 | 0.67 |
| Third-order | 0.24 ± 0.10 | 0.23 ± 0.12 | 0.62 | 0.84 ± 0.39 | 0.58 ± 0.28 | 0.002* |
| Spherical aberration | 0.20 ± 0.08 | 0.23 ± 0.08 | 0.28 | 0.40 ± 0.17 | 0.59 ± 0.17 | <0.001* |

* *P*< 0.05

HOA = high-order aberrations

**Supplementary Table 2.** Univariate Analysis between the Change of Corneal Densitometry after Refractive Surgery and Changes in Corneal Wavefront Aberrations

| Variable | Corneal HOAs | | Corneal coma | | Corneal third-order aberration | | Corneal spherical aberration | |
| --- | --- | --- | --- | --- | --- | --- | --- | --- |
|  | Coef | *P* | Coef | *P* | Coef | *P* | Coef | *P* |
| **Anterior layer** | |  |  |  |  |  |  |  |
| 0 to 2 mm | 0.007 | 0.54 | -0.004 | 0.701 | -0.003 | 0.778 | 0.012 | 0.126 |
| 2 to 6 mm | 0.005 | 0.698 | -0.006 | 0.633 | -0.005 | 0.681 | 0.011 | 0.214 |
| 6 to 10 mm | -0.013 | 0.214 | -0.015 | 0.147 | -0.015 | 0.123 | -0.004 | 0.617 |
| **Central layer** | |  |  |  |  |  |  |  |
| 0 to 2 mm | 0.019 | 0.355 | 0.001 | 0.977 | 0.003 | 0.865 | 0.019 | 0.161 |
| 2 to 6 mm | 0.014 | 0.535 | 0 | 0.989 | 0.002 | 0.938 | 0.013 | 0.399 |
| 6 to 10 mm | -0.025 | 0.15 | -0.027 | 0.114 | -0.027 | 0.094 | -0.009 | 0.453 |
| **Posterior layer** | |  |  |  |  |  |  |  |
| 0 to 2 mm | 0.015 | 0.459 | -0.004 | 0.851 | 0 | 0.994 | 0.011 | 0.394 |
| 2 to 6 mm | 0.01 | 0.636 | -0.008 | 0.709 | -0.004 | 0.852 | 0.009 | 0.521 |
| 6 to 10 mm | -0.022 | 0.227 | -0.032 | 0.086 | -0.03 | 0.081 | -0.005 | 0.705 |
| **Total thickness** | |  |  |  |  |  |  |  |
| 0 to 2 mm | 0.012 | 0.455 | -0.004 | 0.829 | -0.001 | 0.936 | 0.015 | 0.185 |
| 2 to 6 mm | 0.01 | 0.6 | -0.006 | 0.767 | -0.003 | 0.854 | 0.012 | 0.322 |
| 6 to 10 mm | -0.02 | 0.184 | -0.024 | 0.107 | -0.024 | 0.09 | -0.006 | 0.582 |

* *P*< 0.05

HOA = high-order aberrations; Coef = univariate coefficient

**Supplementary Table 3.**  Changes of corneal backscatter after SMILE and FS-LASIK in previous studies

| Reference | Sample size | Measurement | Follow-up | Findings in corneal backscatter^*^ | | |
| --- | --- | --- | --- | --- | --- | --- |
|  |  |  |  | SMILE | FS-LASIK | Comparison |
| (Patel et al., 2007) | 21 patients (42 eyes) with one randomized eye flap creation with a femtosecond laser (IntraLaser FS, CA) and the fellow eye with a mechanical microkeratome (Hansatome, NY) | Custom scatterometer | 1, 3, and 6 months | / | Increased in the anterior and middle thirds of the cornea at 1 and 3 months. | / |
| (Agca et al., 2014) | 30 patients (60 eyes)with one randomized eye with SMILE and the fellow eye with FS-LASIK | In vivo confocal microscopy | 1 week and 1, 3, and 6 months | / | / | The LI at all measured depths (120 and 150μm below epithelium, and 30μm below Bowman’s membrane) and the maximum LI were higher in the SMILE group at 1 week and 1- and 3-month visits but not at 6 months. |
| (Savini et al., 2016) | 23 patients (23 eyes, randomly chosen) with FS-LASIK | Pentacam | 1 day, 1 week, and 1, 3, and 6 months | / | Increased in the 0-10mm zone of anterior layer until 1 month, and the change remained in the 6-10mm annulus until 6 months. | / |
| (Han et al., 2017) | 34 patients (34 eyes, randomly chosen) with SMILE, 40 patients (40 eyes, randomly chosen) with FS-LASIK | Pentacam | 1 day, 1 week, 1, 3, 6 and 12 months, and 3 years | Increased in the 0-6mm zone of anterior layer and the 0-2mm zone of the central layer at 1 day, and decreased in the 0-10mm zone of all three layers at 3 years. | Same as the SMILE group, but also increased at 6-10mm annulus at 1 day. | The increase was higher in SMILE group in the 0-6mm zone of the anterior and central layers. No difference was found at 3 years. |
| (Lazaridis et al., 2017) | 33 patients (58 eyes) with SMILE, 33 patients (58 eyes) with FS-LASIK | Pentacam | 3 months | Decreased in the 0-2mm zone of the central and posterior layers, and 2-6mm zone of the anterior layer. | Decreased in the 0-2mm zone of the central layer, and 0-6mm zone of the posterior layer. | No significant difference. |
| (Pedersen et al., 2017) | 101 patients (101 eyes) with SMILE | Pentacam | 12 months | No significant change | / | / |
| (Poyales et al., 2017) | 109 patients (214 eyes) with SMILE, 74 patients (140 eyes) with FS-LASIK | Pentacam | 3months | Decreased in 10-12mm annulus. Decreased in the anterior layer and total corneal thickness. | Decreased in 10-12mm annulus. | No significant difference. |
| (Shajari et al., 2018) | 47 patients (89 eyes) with SMILE, 30 patients (57 eyes) with FS-LASIK | Pentacam | 1 week, 1, 3, 6, and 12 months | / | / | No significant difference after Holm-Bonferroni Sequential correction. |

LI: light intensity.

^*^ all *p*< 0.05; for Pentacam, the anterior layer was defined as the anterior 120μm of the cornea, the posterior layer as the posterior 60 μm, and the central layer as the cornea at mid-distance between the two layers.
